# The times they aren’t a-changin?

**DOI:** 10.1007/s44470-026-00227-z

**Published:** 2026-11-05

**Authors:** Karin G. Johnson, Sabra M. Abbott

**Affiliations:** 1 Department of Neurology, UMass Chan School of Medicine-Baystate, Springfield, MA USA; 2 Department of Neurology, Northwestern University, Chicago, IL USA

Social jet lag, the idea of sleeping later on free days compared to work days, is a marker of circadian disruption that has been associated with multiple negative outcomes. It represents a mismatch between endogenous circadian time, which often more closely follows solar time, and exogenous social time, which is set by external responsibilities. Early work, school or care-giving schedules, set by the social clock, force early awakenings, while endogenous circadian timing makes it more difficult to fall asleep at the earlier times required during the week.

In their article recently published in the *Journal of Clinical Sleep Medicine*, Fu et al. examine the impact of social jet lag across 63,752 adults living across China, utilizing a photoplesmography-based wearable device [1]. China is unique because it spans the distance of 5 time zones using a single clock time. This provides a unique opportunity to evaluate the impact of setting social clocks in misalignment from solar timing. This mirrors what often occurs elsewhere in the world during daylight saving time. During daylight saving time, sunrises and sunsets occur at a later social time, which is similar to the mismatch observed for those living further west within the same time zone.

Daylight saving time impacts sleep from both ends. By moving the social clock later, individuals are exposed to more light in the evening, increasing the likelihood of circadian delays making it harder to fall asleep at the required time. In the morning, the mismatched social timing often requires waking prior to the endogenous circadian wake time, leading to increased morning grogginess. Later sunrises with respect to social time lead to decreased exposure to morning light at required wake times, which normally helps provide a daily advancing signal. Morning light is especially important in evening chronotypes and teenagers who are most reliant on these daily signals to prevent their rhythms from delaying further.

In a natural experiment, Russia tried permanent daytime saving time before changing to permanent standard time [2]. Borisenkov et al. found that adolescents were 7–8% more likely to experience at least 1 h of social jet lag and 17% more likely to experience at least 2 h of social jet lag when exposed to seasonal or permanent daylight saving time compared to permanent standard time. Despite similar sleep duration, they found an increased incidence of seasonal depression suggesting the circadian disruption can affect outcomes separate from sleep duration. When discussing permanent daylight saving time, a common argument made is that later school start times could mitigate the negative effects of permanent daylight saving time on adolescents. However, if we were to adopt permanent daylight saving time, the 8:30 AM recommendation for high school start would need to shift to 9:30 AM to gain the same benefits currently seen with later start times.

In Fu’s study, those individuals living further west in the time zone had later sleep timing and greater social jet lag. In addition, they found high social jet lag was associated with smoking, caffeine use, and alcohol use. This aligns with other studies linking either social jet lag or early school start times with an increase in risk taking behaviors [3]. Fu et al. also found a correlation with increased rates of self-reported depression, diabetes, and obesity. Other studies looking at daylight saving time and an individual’s geographic position in the time zone have demonstrated similar findings [4, 5]. Clock misalignment with later sunrise and sunsets, as would be seen during daylight saving time or living on the western edge of the time zone, are associated with higher rates of cancer [6] stroke [5] suicide [7] fatal motor vehicle accidents [8], and lower academic achievement [9].

A common myth used to support permanent daytime saving time is that later sunsets will promote an increase in exercise and thus better health. However, Fu’s study found that higher social jet lag was associated with lower step attainment and WHO-active status. This aligns several studies that have shown no increase in overall exercise levels with daylight saving time especially in the USA [10, 11].

The American Academy of Sleep Medicine and over 100 medical and scientific societies have endorsed permanent standard time as the best way to end seasonal clock change. Fu et al.’s study provides further data to support the negative effects of chronically misaligning solar and social clocks, as would occur with permanent daylight saving time.

## Data Availability

Not applicable.
